# Embedded Distributed Optical Fiber Sensors in Reinforced Concrete Structures—A Case Study

**DOI:** 10.3390/s18040980

**Published:** 2018-03-26

**Authors:** António Barrias, Joan R. Casas, Sergi Villalba

**Affiliations:** 1 Department of Civil and Environmental Engineering, Technical University of Catalonia (UPC), c/Jordi Girona 1-3, 08034 Barcelona, Spain; joan.ramon.casas@upc.edu; 2 Department of Engineering and Construction Projects, Technical University of Catalonia (UPC), c/Colom 11, Ed. TR5, 08022 Terrassa, Spain; sergi.villalba@upc.edu

**Keywords:** fiber optics, structural health monitoring, distributed fiber sensing, civil engineering, load testing, reinforced concrete, cracking

## Abstract

When using distributed optical fiber sensors (DOFS) on reinforced concrete structures, a compromise must be achieved between the protection requirements and robustness of the sensor deployment and the accuracy of the measurements both in the uncracked and cracked stages and under loading, unloading and reloading processes. With this in mind the authors have carried out an experiment where polyimide-coated DOFS were installed on two concrete beams, both embedded in the rebar elements and also bonded to the concrete surface. The specimens were subjected to a three-point load test where after cracking, they are unloaded and reloaded again to assess the capability of the sensor when applied to a real loading scenarios in concrete structures. Rayleigh Optical Frequency Domain Reflectometry (OFDR) was used as the most suitable technique for crack detection in reinforced concrete elements. To verify the reliability and accuracy of the DOFS measurements, additional strain gauges were also installed at three locations along the rebar. The results show the feasibility of using a thin coated polyimide DOFS directly bonded on the reinforcing bar without the need of indention or mechanization. A proposal for a Spectral Shift Quality (SSQ) threshold is also obtained and proposed for future works when using polyimide-coated DOFS bonded to rebars with cyanoacrylate adhesive.

## 1. Introduction

### 1.1. Structural Health Monitoring (SHM) 

The decay of the present network of civil engineering infrastructures and the consequent extension of their lifetime period is a highly imperative subject in today’s society. Critical infrastructures such as bridges, dams, buildings, tunnels and power plants are inevitably subject to deterioration, which greatly hinders their expected performance with high economic consequences and can even endanger the safety of human lives.

In order to prevent and overcome this circumstance it is essential for infrastructure management owners to correctly maintain and rehabilitate the structural condition of these structures over time. This can be achieved by the installation of a set of sensors that follow the structures’ behaviour and detect abnormalities allowing for the identification of damage. This enables a quick and preventive deployment of low-cost minor repairs that can prevent expensive or even irredeemable repairs and/or strengthening. This procedure is known as the Structural Health Monitoring (SHM) practice.

Additionally, this practice is not restricted to outdated or ageing structures. Effectively, when dealing with new and relevant structures the installation of sensors during the construction phase must be well planned and conducted since it provides an immense aid in detecting early stage degradation and damage [1].

Moreover, when deployed correctly, a SHM system provides important quantitative information on the state of the structure, which is simultaneously convenient for the structure’s owner and for structural designers by providing important and real world structural behaviour data for future designs.

This topic has been developed and researched with great interest in the last two decades but unfortunately has yet to be applied in a standardized and efficient way since there is still an important lack of reliable and relatively economical SHM civil engineering solutions [2].

### 1.2. SHM with Distributed Optical Fiber Sensors (DOFS)

Conventionally, the overall condition assessment of a civil structure is carried out by well-trained engineers through visual inspection. This is a very subjective practice, which is dependent on individual experience and background in safety condition assessment. With the initial introduction of SHM systems part of this subjectivity may be eliminated. Nevertheless, the reliability and robustness of a health monitoring system is strongly correlated to the accuracy and robustness of the sensors deployed for each application and the posterior analysis of the gathered data.

In an initial stage and until very recently, the most regularly practiced SHM approaches were based on electrically powered sensors, such as strain gauges, inclinometers, displacement transducers, accelerometers and so on. However, these sensors when applied to real world conditions present significant shortcomings which may hamper the use of the obtained data [3]. 

In this sense, the use of optical fiber technology has been gaining popularity in the past two decades as one of the most researched topics within SHM sensors. Some inherent advantages, that optical fiber sensors (OFS) present when compared with their electrical based counterparts, are the fact that they are resistant to electromagnetic interference, durability, immunity to corrosion, good performance within large temperature fluctuations and their small size and lightweight. [4].

Within these type of sensors, the most popular applications were made through the use of discrete or quasi-distributed Fiber Bragg Grating (FBG) sensors [5] and have been extensively discussed in different publications in past years [6,7,8,9,10].

In addition, standard monitoring practice is generally based in the use of a reduced number of discrete or point sensors which are supposed to be representative of the global structural behavior [11]. Although the use of discrete sensors delivers interesting data of the monitored specimen related to its local behavior, relevant information of the global structure’s performance might be absent from the acquired data. This is important in several cases, especially in the case of SHM applications deployed in large-scale structures such as bridges, high-rise buildings, tunnels, power plants and dams. Furthermore, in this type of structures, the number of point sensors (and its corresponding connecting cables) required to perform a suitable global monitoring can increase exponentially, which consequently escalates the associated costs and technical challenges for effective deployment.

Moreover, especially for the case of heterogeneous materials such as concrete, the exact prevision of the damage location (normally associated with cracking and corrosion) is practically impossible. Therefore, with the use of discrete sensors there is a higher probability of not detecting the formation of cracks in these structures. 

**Figure 1:**
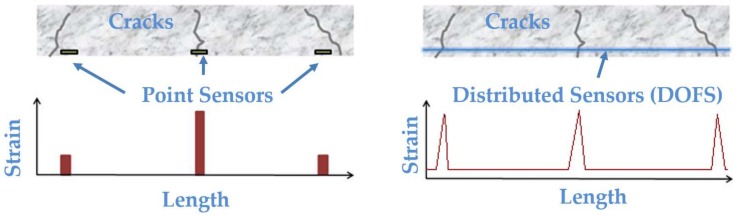
Spatial distribution detection with OFS (**left**) and DOFS (**right**).

**Figure 2:**
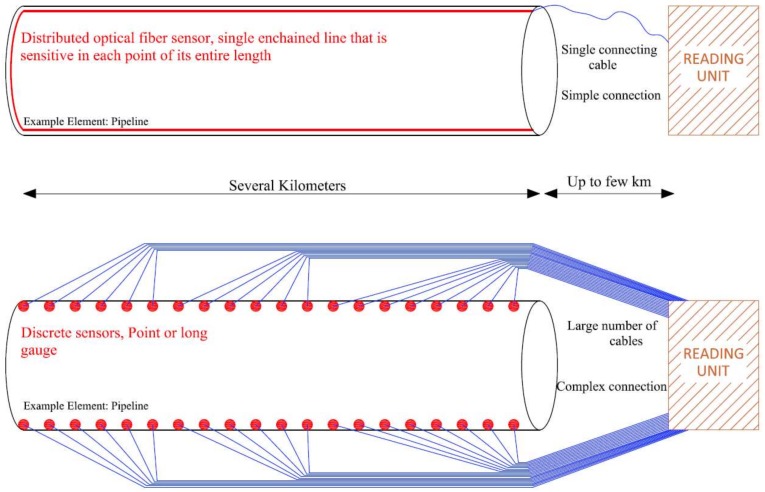
Comparison of use of distributed sensor (**top**) and point sensors (**bottom**) in large scale structures [13].

In this context, Distributed Optical Fiber Sensors (DOFS), while sharing the main advantages and capabilities of the aforementioned OFS, offer an additional and exclusive advantage over them as becomes evident in Figure 1 and Figure 2 concerning crack detection capabilities and simplification of multiplexing.

Apart from DOFS technology, the development of other distributed sensing techniques and their use for crack detection on reinforced concrete structures has observed significant progress in recent years, such as the research of smart concrete elements. This are cement-based materials doped with conductive carbon nanoinclusions [12].

DOFS are able to measure strain and/or temperature along their entire length by means of light scattering, which allows for the monitoring of virtually every cross-section of the structural element.

Hence, the scattering phenomenon is fundamentally characterized by the interaction between the light emitted and subsequent propagation and the physical medium where this occurs. More so, three different types of scattering processes may occur in a DOF sensor, which are the Raman, Brillouin and Rayleigh scattering, where each presents advantageous or limited capabilities depending on the objectives and specificity of each monitoring solution.

For instance, Raman scattering is characterized by high dependence on temperature which has found some successful applications in the civil engineering field [14] but has mostly been applied in other areas such as art restoration [15] and forensics [16].

In contrast, the Brillouin scattering-based DOFS have been the most studied and practiced in civil engineering structures, mainly due to their extended measurement range capability, which can go up to several kilometers. In this way, these type of sensors have been used to a great extent for long-distance distributed strain and temperature measurements in structural and geotechnical monitoring [17]. However, the achievable spatial resolution of these sensors (around 1 m) without the use of any complex post-processing algorithms is rather limited, which is not ideal for local damage detection and localization. 

Nevertheless, important advances and interesting applications have been conducted recently with the use of Brillouin-based scattering DOFS. From research on crack monitoring in concrete pavements [18], surface microcracks detection and monitoring [19], shrinkage induced delamination detection for ultra-high performance concrete (UHPC) [20] and geotechnical engineering applications in the monitoring of the changes of the concrete curing temperature profile of piles [21] and soil slope during infiltration monitoring [22].

Finally, Rayleigh scattering, although limited to a sensing range of around 70 m, provides a substantially higher spatial resolution of 1 mm through the use of the Rayleigh backscattering Optical Frequency Domain Reflectometry (OFDR) [23], which is appropriate for the monitoring of crack formations in concrete structures. A more detailed description of the OFDR sensing mechanism can be consulted in [24].

**Figure 3:**
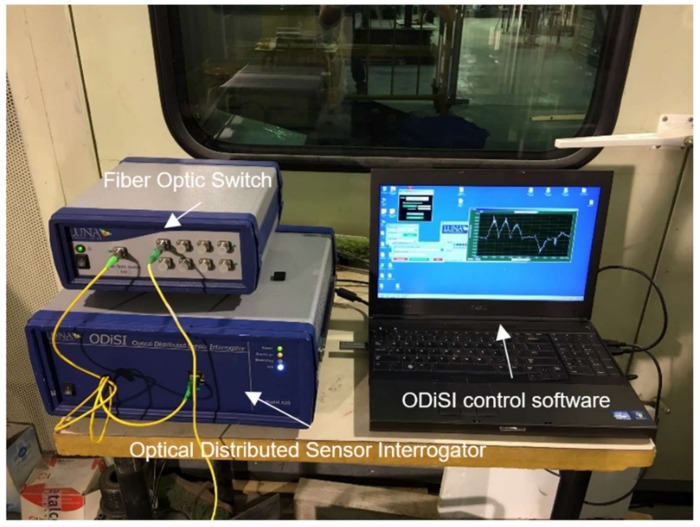
ODiSI A system used in this research case study.

As a result, this later scattering technique is the one deployed and researched in this study, aimed to the monitoring of reinforced concrete structures, through the use of the commercial optical interrogator ODiSI A from LUNA Technologies (Roanoke, VA, USA), Figure 3. This system has an accuracy and repeatability of ±2 µε, ±0.2 °C, a measurement range of −50 to 300 °C and ±13,000 µε. Furthermore, this system enables a user-controlled sub-cm spatial resolution and a maximum sensing length of 50 m [25].

With this sensor, strain or temperature measurements are achieved by initially measuring and storing the Rayleigh scatter signature of the optical fiber installed in the structural element and being read by the ODiSI interrogator at an ambient state, constituting in this way the baseline measurement. This interrogator collects in the spectral frequency domain the light backscattered from the fiber sensor. This backscattered spectrum although seemingly random is stable and repeatable thus establishing a fingerprint of the DOFS.

Later, the scatter profile is measured after the variation of strain or temperature at any point along the length of the fiber. These two data sets are then cross correlated at the sensor locations to determine the spectral shift of the scattered light [26]. The shift in the spectrum is directly correlated with the strain or temperature variation which through the use of calibration constants enables the use of this scattering spectral shift as a monitoring sensing technique.

Up to now, some laboratory tests were carried out using also Rayleigh OFDR based DOFS both bonded in the concrete surface and embedded in reinforcing bars into the concrete. However, in all these tests, the application of monotonically increasing load was carried out, as their mean objective was to study the feasibility of assessing corrosion by means of distributed sensing. In the present study, the performance of the sensor bonded to the concrete surface and to the reinforcing bar in cyclic loading in cracked condition is investigated for the first time.

## 2. Laboratory Experiments

### 2.1. Motivation

Notwithstanding all the advantages of the use of DOFS-based technology in SHM applications, this is still a relatively recent field of study which requires a significant research and development effort when applied to a complex and heterogeneous material such as reinforced concrete. In this line, one of the key challenges on the use of these sensors is the existing compromise between the accuracy in measuring the strain data from the monitored material and the achievable protection and durability of such a sensor when exposed to the natural environmental conditions in real world applications [27]. 

When applying these sensors, it is easily understandable that a relatively thicker coating of the fiber itself provides a higher protection, hence, benefiting handling and manipulation at the time of installation, reducing the probability of rupturing, and also providing protection against external disruptive agents during the lifetime period. Nevertheless, the thicker the coating, the greater the effect on the measured data, thus affecting and clouding the real developed strain of the monitored specimen. On the other hand, a thinner coating provides a higher fragility and probability of rupturing when handling it despite the significantly increase of the effectiveness of the strain transfer between sensor and material. 

Different studies have been made regarding the coating influence on the strain transfer between the sensor and the host material on both discrete [28,29] and distributed optical fiber sensors [30] by way of assessing the difference between the apparent and actual strain profile. This aspect is even more relevant for applications in reinforced concrete structures. Due to the heterogeneity of the different materials (aggregates of different size and type, cement paste) that constitute the concrete material and the roughness of its surface, a smooth and optimal bonding of the sensor to the material is difficult to achieve. Therefore, a smoothing and cleaning of the original surface is mandatory [31,32].

During the conduction of a comprehensive literature review on the use of Rayleigh OFDR-based DOFS in civil engineering SHM applications, the authors observed some studies where to overcome this mentioned challenge, DOFS were not bonded, as usual, to the surface of the concrete specimen, but rather directly attached to the rebars of a reinforced concrete element [33]. Here the argument is made that the sensor is better protected from environmental conditions during its lifetime due to this implementation method. Notwithstanding, in this case it is still necessary to previously mechanize the rebar where the fiber was to be bonded, what can be a tiresome and cost consuming operation while also having some effect in the accuracy of the results. Moreover, a still relatively thick coating was used, not fully taking advantage of this new improved condition for the fiber durability.

Another group of researchers also using Rayleigh backscattering bonded the fiber directly on the rebars without previous mechanization. The sensor was installed internally in reinforced concrete elements in order to detect pitting corrosion in these elements [34]. Here, polyimide coated fibers were implemented to the rebar elements of embedded reinforced concrete beams under four-point bending tests showing promise for detecting localized corrosion. 

Later, this group continued this research with a test where distributed sensing was used to assess the impact of corrosion on bond performance in reinforced concrete [35]. Here, tensile tests were conducted on bare reinforcement and reinforced concrete specimens subjected to accelerated corrosion where polyimide and nylon coated DOFS were implemented to rebars. This technology was able to measure the corrosion effects on the concrete and steel bond as well as locate areas of pitting corrosion that were visually hidden by the surrounding concrete. 

These same authors followed this research with an experiment where six reinforced concrete beams under varying levels of corrosion were instrumented with DOFS and tested in three-point bending [36]. The used distributed sensing technology was again able to detect the effect of the corrosion on the loss of bond between the concrete and reinforcement.

Taking this into account, the present paper aims to further analyse the performance and feasibility of deploying a single thin polyimide coated low bend loss fiber (total diameter of core plus coating of 155 µm [37]) to rebar elements without any preliminary mechanization of the steel. However, in the present case, the main objective is not related to the ability of the sensor in the detection of corrosion induced damage, but to examine the performance and feasibility of the DOFS when the sensor is crossing a crack and the crack is opening and closing, what may create a risky scenario for damaging the fiber taking into account the roughness present in the crack lips.

The absence of rebar mechanization simplifies the installation process, decreases the associated costs and increases the reliability of the measurements as almost not coating material is interposed between the reinforcing bar and the DOFS. In a first step, the accuracy of the fiber optic bonded to the rebar is examined and afterwards, the performance of the sensor in measuring the strain in the steel when the rebar is embedded into the concrete is checked for both loading and unloading scenarios that may challenge the fiber integrity and accuracy. Moreover, in this later step, the same optical fiber is used for both the measurements in the rebar as well as the concrete surface.

### 2.2. Tensile Test on an Isolated Rebar

The objective of this simple test in a single rebar is to observe the performance of the method of bonding the thin coated DOFS directly to the steel without mechanizing it before as done in other experimental works [33]. The fiber optic used in the test is of polyimide type.

#### 2.2.1. Description

**Figure 4:**
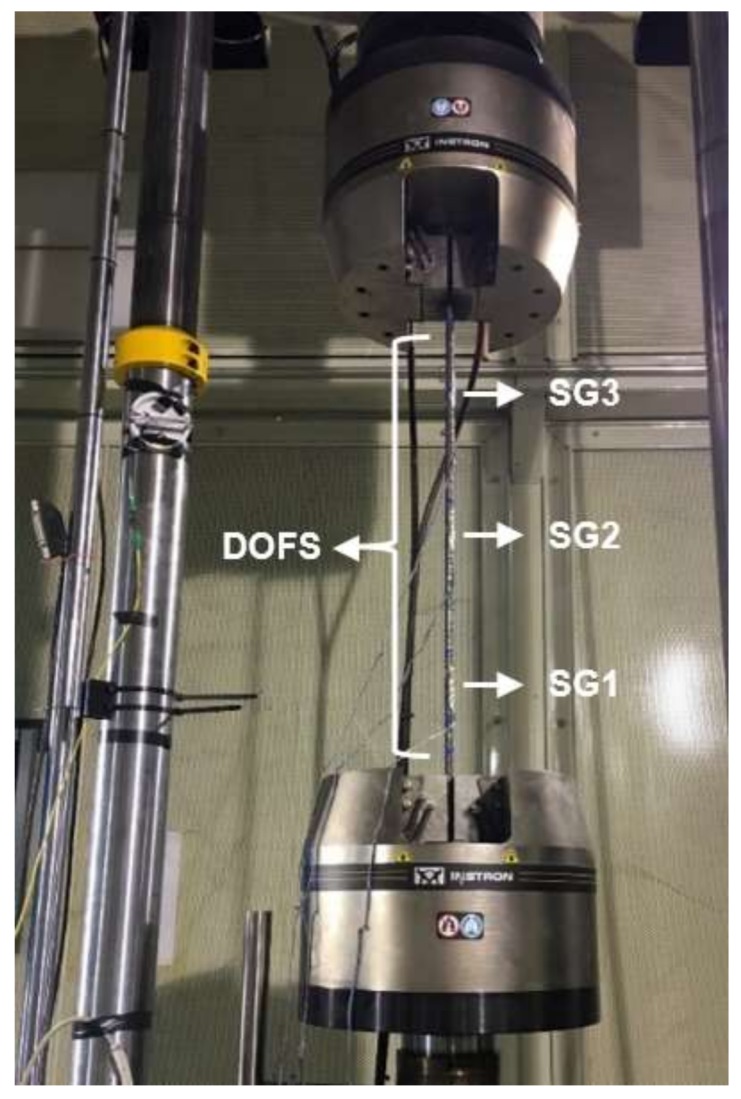
General scheme of the instrumented rebar for the tensile test with the DOFS and the strain gauges.

A thin-coated polyimide fiber was adhered for nearly 75 cm of its length to the longitudinal lateral rib of a single Φ12 (12 mm of diameter) S500 standard rugged steel rebar through the use a two-component epoxy as the adhesive. In addition, three electrical strain gauges were implemented sparsely on the other side of the rebar (in order to minimize the interference with the DOFS measurements) for comparison purposes. The strain gauge SG2 was positioned at the center of the rebar and the remaining two (SG1 and SG3), 22.5 cm spaced to each side of SG2 (Figure 4).

The induced tensile stress in the rebar was limited to the elastic range of the steel as this rebar had to be used later embedded in the concrete during the three-point bending test. Consequently, two-different load stages were applied to the specimen through the hydraulic press seen in Figure 4. These load stages were of 11.3 kN and 22.6 kN expected to produce tensile stresses of 100 and 200 MPa respectively, thus far below the yielding stress of the steel.

#### 2.2.2. Results

**Figure 5:**
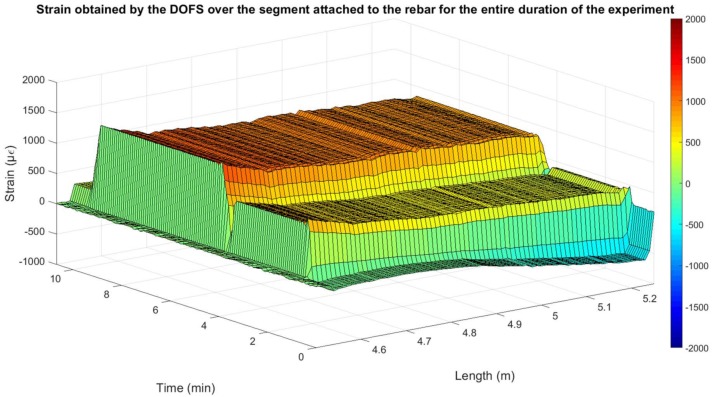
DOFS measured strain for rebar tensile test.

Figure 5 represents the strain measured by the DOFS during this load test. One thing that is important to mention is that the length of the DOFS was 5.2 m, but only the last 0.75 m were adhered to the rebar during this test and this is the part shown in the figure.

The two strain levels produced by the two mentioned load levels are clearly seen in Figure 5. In addition, the distribution of the strain over the entire length of the monitored rebar due to the distributed capability of the DOFS is observable. As expected, according to the cross-sectional area and elasticity modulus of the rebar, these strains are close to 500 µε for the first load stage of 11.3 kN and to 1000 µε for the second load stage of 22.6 kN.

**Figure 6:**
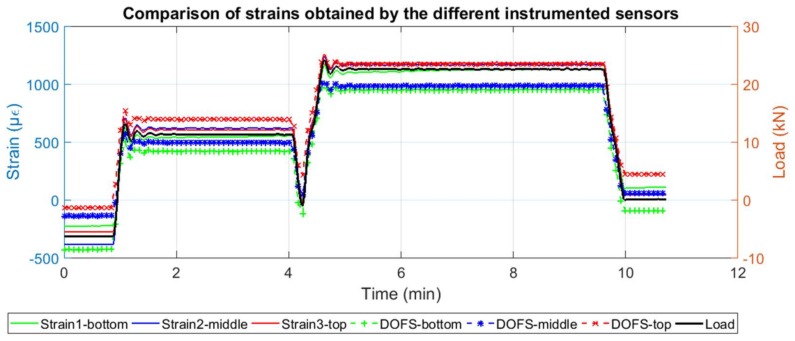
Comparison of strains obtained by the two different sets of deployed sensors.

In Figure 6, the strain values measured by the strain gauges over time are compared to the ones obtained by the DOFS at their respective point locations. Here, a good agreement of both set of sensors is observable especially represented at the transition between unloaded and loaded stages.

It is also observable how the strain measured by both sets of sensors is slightly higher on the top part of the rebar compared to the other values measured at lower points. One possible justification for this is the fact that while performing the tensile test, the top jack introduces tensile tension by pulling the top part of the rebar. It is observed that this variation is higher in the DOFS than it is in the deployed strain gauges.

Nonetheless, the obtained results give confidence for the use of DOFS attached directly to the rebar without the need of a previous mechanization of the steel and a thick plastic coating of the fiber. The results clearly show that the DOFS is measuring the actual strain in the steel, as confirmed by the strain gauges, without the need to any further correction due to the interposition of the coating. Once the correct performance of the deployment method in the rebar was checked, the next step was to look at its performance when embedded into concrete, both before and after cracking of the specimen and under loading and unloading conditions.

### 2.3. Load Test on Reinforced Concrete Beams

**Figure 7:**
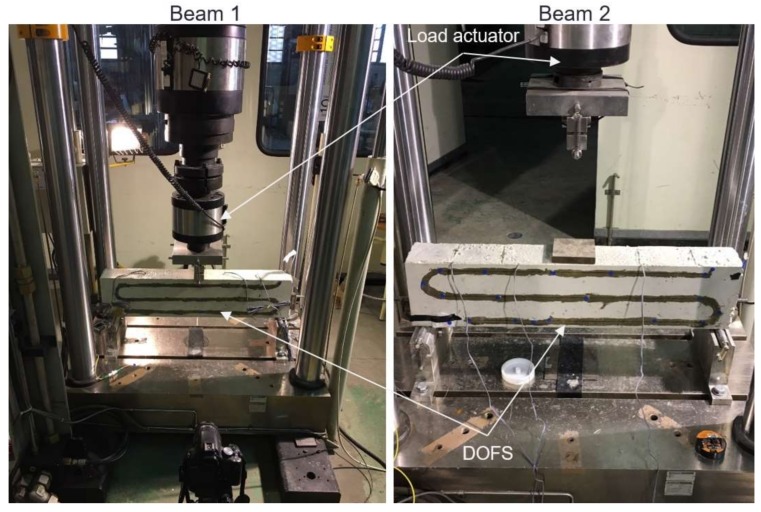
Reinforced concrete beam specimen 1 (**left**) and 2 (**right**).

As a result, and after evaluating the performance of attaching very thin polyimide coated DOFS directly to a rebar for strain measuring, it was decided to perform a three-point load test on two different reinforced concrete beams specimens represented in Figure 7. 

#### 2.3.1. Description 

**Figure 8:**
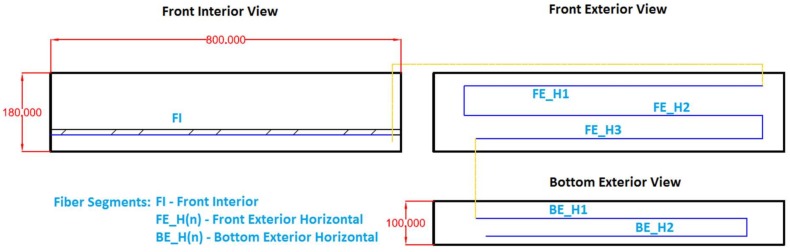
Definition scheme of DOFS deployed on each beam—all dimensions [mm].

The two reinforced concrete beam specimens present a cross-section of 100 mm width and 180 mm height. The total length was 800 mm. Only one reinforcing bar was deployed at the center of the cross-section and as the rebar used in the tensile test, it was a 900 mm long Φ12 S500 rebar. The concrete cover was 40 mm. Since both DOFS deployed in this test had a 5.2 m length, it was decided to take advantage of the entire length and install the rest of them at the surface of the concrete after its hardening. These segments and the dimensions of the beams are depicted in Figure 8. The only difference between the two specimens was the bonding material of the fiber to the rebar (cyanoacrylate and two-component epoxy). The same two-component epoxy adhesive is used in both specimens to bond the fiber to the concrete surface.

With this scheme, it is possible to measure simultaneously the strains both at the rebar and the surface of the concrete using a single sensor. Although the used system allows the user to define sub-cm spatial resolution, it was decided to conduct the measurements of these load tests using a 1 cm spatial resolution, since from other experiences the authors concluded that using a higher spatial resolution provides no practical benefit when applied to concrete structures. This allowed for the measurement of more than 500 different points with each DOFS. The measurements were made with a 5 s interval.

**Figure 9:**
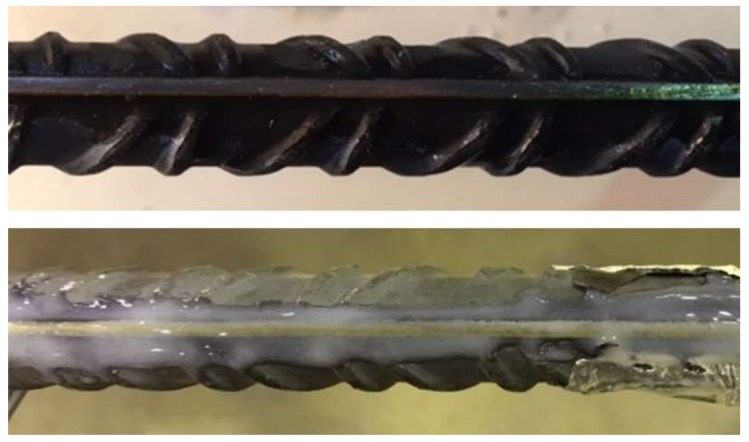
Adhesives on rebar of each beam specimen: cyanoacrylate (Beam 1—**top**), two-component epoxy (Beam 2—**bottom**).

For the bonding of the DOFS to the rebar segment, two different adhesives were used in each specimen. In the first beam, a cyanoacrylate adhesive was used whereas in the second beam the installation of the sensor was performed with a two-component epoxy (Figure 9). The idea is to assess which adhesive performed better in these conditions. Finally, as in the case of the tensile rebar test, three strain gauges were installed in the rebar of both specimens for comparison purposes.

Due to the thin coating of the sensors and their subsequent fragility, it was necessary to have great care when pouring the concrete into the moulding where the full set of sensor plus rebar was installed. During concreting, the final part of the fiber not bonded to the rebar was protected with small plastic tubes and fixed to the interior lateral part of the moulding as shown in Figure 7. 

After the hardening of the concrete, the remaining length of the fiber was bonded to the surface with the pattern seen in Figure 7 and Figure 8. This scheme was laid out in a way that it was possible to measure compression strains at the top lateral segment (FE-H1); values close to zero at the neutral axis in the middle lateral segment (FE-H2); tension strains at the bottom lateral segment (FE-H3) and the two bottom exterior segments (BE-H1 and BE-H2). For the bonding of the external DOFS segments to the concrete, the two-component epoxy adhesive was used based in successful past experiences of the authors [27,31,32].

With these external segments, it was also expected to detect and follow the evolution of cracking. For this, at the time of pouring the concrete, additional cylindrical samples were also cast. They were later tested at the same dates of the tests in the beam specimens in order to obtain the mechanical properties of the concrete as summarized in Table 1. Here, $f_{cm}$ is the concrete mean compressive strength, $f_{ctm}$ the concrete mean tensile strength, *E* is elasticity modulus and $\varepsilon_{fct}$ the expected maximum tensile strain before concrete cracking. According to these values, the expected cracking load was 8.86 kN in beam 1 and 10.15 kN in beam 2.

**Figure 10:**
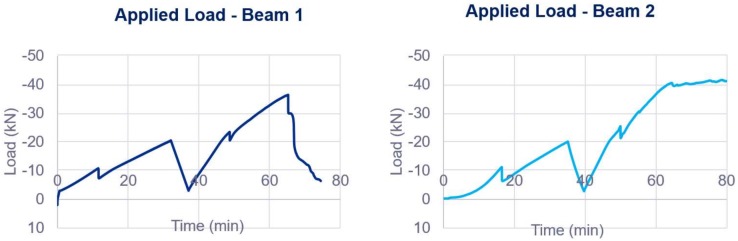
Applied load to each beam specimen.

The goal of the tests was to load the beams up to a load much higher than the cracking load and unload again to observe the performance of the sensor after the occurrence of damage and the further reloading. The loading scheme for each specimen is shown in Figure 10.

#### 2.3.2. Results—Beam 1

**Figure 11:**
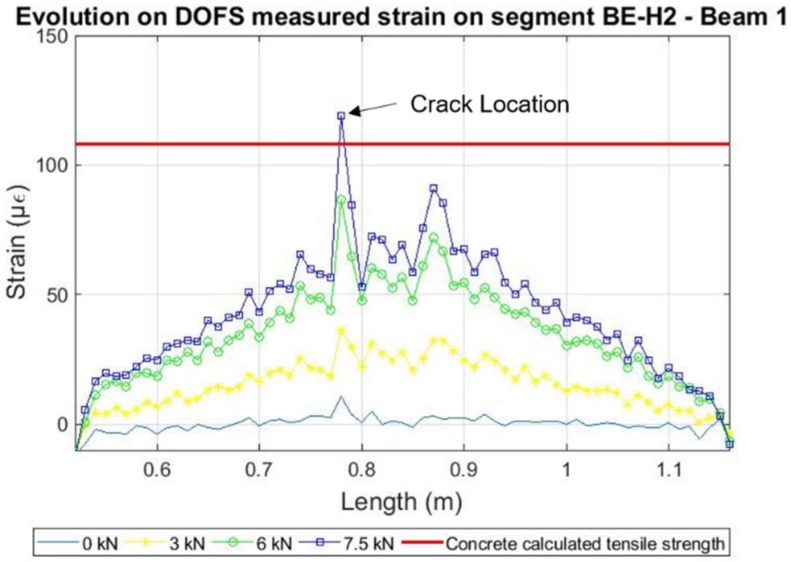
Detection of cracking at the surface of the concrete in beam 1.

In this beam, according to the DOFS measurements, the expected concrete tensile strength capacity of 108 µε was surpassed for a load of 7.5 kN as seen in Figure 11. However, although being detected by the DOFS, the crack was not yet visually observable. It is not until the load of 10.81 kN which corresponds to minute 11.6 that damage is detected at the segment deployed in the rebar. Moreover, the sensor stopped working around the 51 min mark for a load of 24.3 kN during the second loading stage.

**Figure 12:**
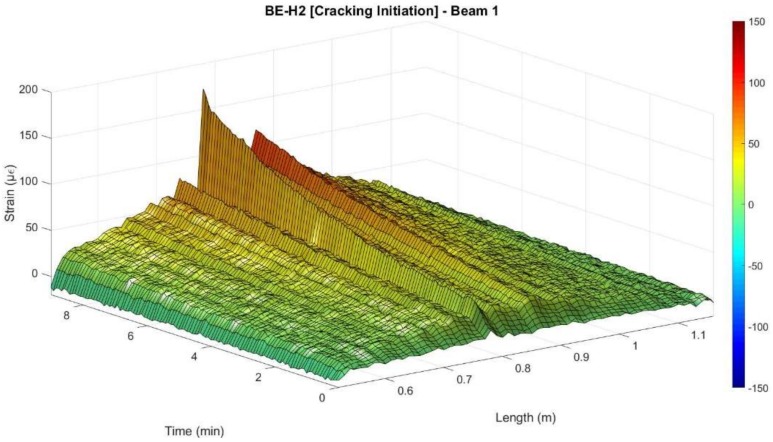
Evolution of strains verified for segment BE-H2 of Beam 1.

Notwithstanding, it was also possible to detect how after the development of cracking the measured values of the DOFS at the location of the crack presented magnitude peaks of either positive or negative values, indicating an invalid measurement. This behaviour after cracking was especially noticeable in the segment attached to the rebar (FI) and will be further discussed in Section 3. However, when analysing each individual segment before the occurrence of cracking, it is possible to observe how the measurements follow smoothly the developed strains as seen in Figure 12.

#### 2.3.3. Results—Beam 2

**Figure 13:**
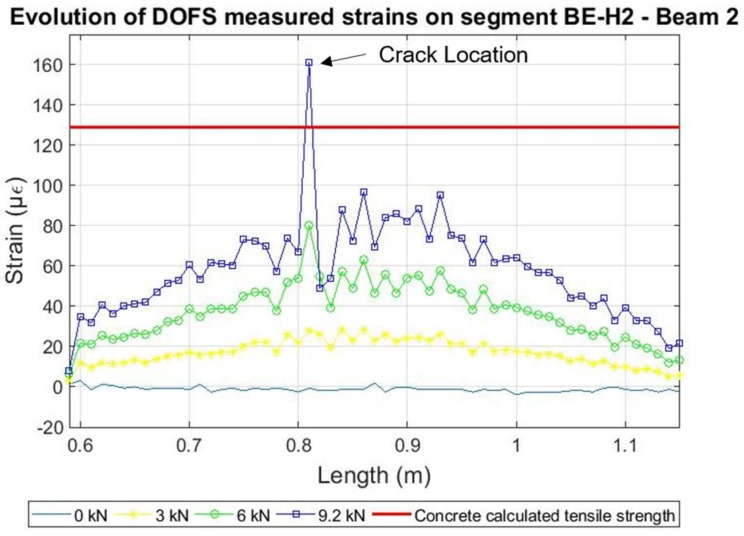
Detection of cracking at the surface of the concrete in beam 2.

Here, according to the DOFS measurements, the expected concrete tensile strength of 129 µε was surpassed for a load of 9.2 kN as detected by the segments adhered to the bottom surface of the concrete as seen in Figure 13. However, as in beam 1, it was not until the minute 16.4 that the damage was detected at the rebar for a load of 11.23 kN.

**Figure 14:**
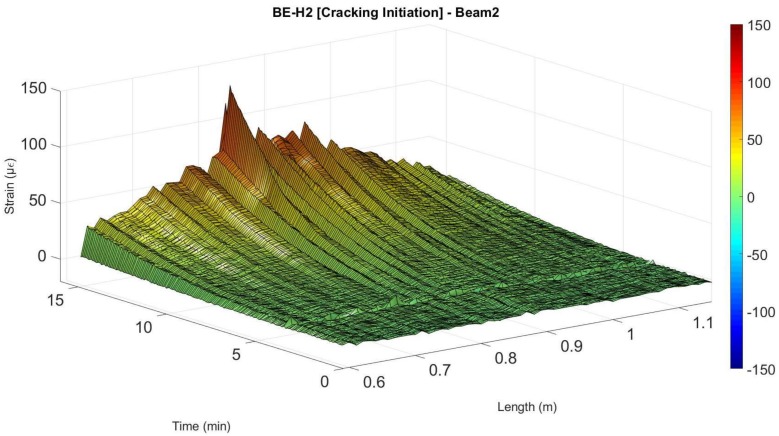
Evolution of strains verified for segment BE-H2 of Beam 2.

Nevertheless, after the development of cracking the DOFS measured strains at the location of the damage show the same alternate positive and negative peaks as obtained in beam 1. Afterwards, the sensor started measuring very inaccurate data starting around the minute 32 for most of the length of the fiber, which corresponded to a load of 17.8 kN until breaking of the sensor close to minute 45. Notwithstanding, for all effects, the fiber sensor was considered unreliable from the minute 32. As before, it is however possible to follow smoothly the evolution of the developed strain along the pre-cracking scenario as seen in Figure 14.

#### 2.3.4. Comparison with Strain Gauges

**Figure 15:**
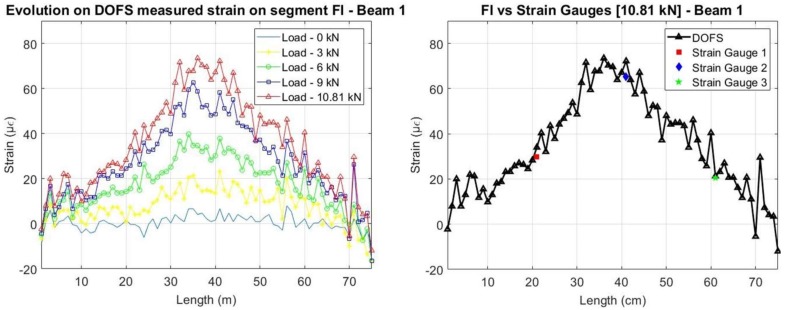
Strains measured by the DOFS on the embedded segment for Beam 1.

**Figure 16:**
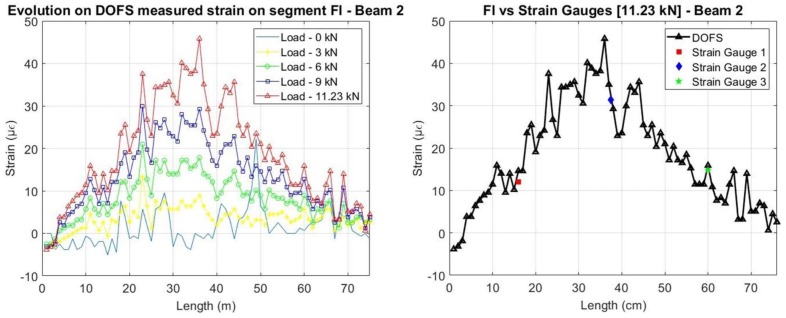
Strains measured by the DOFS on the embedded segment for Beam 2.

Regarding the segment adhered to the rebar, it is possible to compare the DOFS strains with the ones obtained by the strain gauges. As it is possible to see from Figure 15 and Figure 16, before the development of cracking, a great agreement between the two sets of sensors is achieved. Notwithstanding, it is possible to observe how the measurements in Beam 1 (cyanoacrylate bonded) present an overall smoother reading for the initial unloaded stage when compared with Beam 2 (epoxy bonded).

In both beams, the two set of sensors measure, as expected, higher strains at mid-span. It is also possible to see, as mentioned before, that for the load levels where cracking has already occurred at the concrete surface, the damage is not yet detected at the rebar level.

After the development of cracking, due to the aforementioned high magnitude peaks observed for the measurements obtained in this segment and also due to the distributed capacity of the DOFS, the comparison of these two set of sensors is hard to achieve and will be only possible after a post-processing of the raw data as presented in Section 3.

As a result, it is necessary to overcome the challenge presented by the inaccurate measurements after the occurrence of cracking in order to better analyse the obtained data in this stage. One possible way to do it is by removing the inaccurate measurements and interpolating based on the remaining accurate values. In order to assess which values are accurate or not, the spectral shift quality (SSQ) of the measured data has to be analysed.

## 3. Post-Processing

### 3.1. Spectral Shift Quality—SSQ

When analysing monitored data acquired through the use of optical frequency domain reflectometry (OFDR) based DOFS, such as the ODiSI A system used in this experiment, it is always important to evaluate and assure the Spectral Shift Quality (SSQ) of the obtained measurements.

According to the manufacturer of this data acquisition system (LUNA Technologies), the SSQ is a qualitative measure of the strength of the correlation between the conducted measurement (at any point and time) and the original baseline reflected spectra [26]. This is calculated as shown in the following expression:

(1) $$\mathrm{Spectral}\;\mathrm{Shift}\;\mathrm{Quality}=\frac{\max\left(U_{j}\left(\upsilon \right)⋆U_{j}\left(\upsilon -\Delta \upsilon_{j}\right)\right)}{\Sigma U_{j}{\left(\upsilon \right)}^{2}}$$

■ $U_{j}\left(\upsilon \right)$ is the baseline spectrum for a given segment of data;
■ Uj(υ−Δυj) is the measurement spectrum under a strain or temperature change;
■ ⋆ symbol is used to represent the cross-correlation operator.

In this way, the SSQ is the maximum value of the cross-correlation of the baseline measurement and measurement spectra normalized by the maximum expected value. Hence, the value of the SSQ should theoretically be between 0 and 1, where 1 is obtained when a perfect correlation is achieved and 0 when it is uncorrelated. This value can also, in practical terms, be observed to be above the value 1 due to small variations in the laser power.

**Figure 17:**
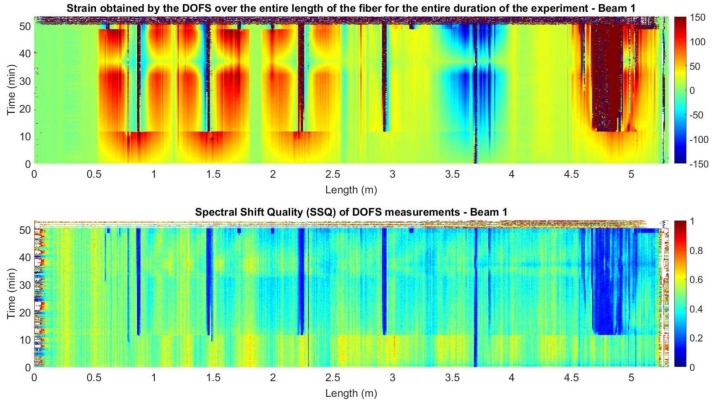
Strain and SSQ for beam 1.

**Figure 18:**
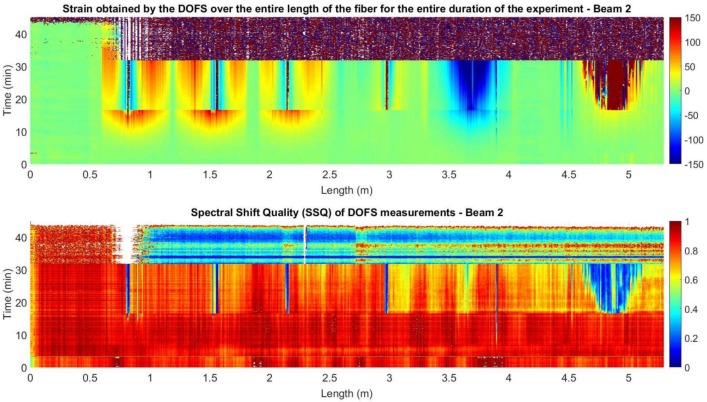
Strain and SSQ for beam 2.

Therefore, the manufacturer advises to disregard any measurements with a SSQ below 0.15, since when this threshold is reached, it is likely that the strain or temperature change has exceeded the measurable range. This also implies, that the further the damage is developed in the monitored structural element, the lower the SSQ values will be. In this way, when calculating the values SSQ for the measurements obtained in the two beams, Figure 17 and Figure 18 are obtained.

The analysis of Figure 17 and Figure 18 shows how the locations of the values with an SSQ below 0.15 and the strain values with high magnitude peaks are greatly correlated. 

This situation of the dropping of the SSQ values during flexural load tests is also reported in [38]. In fact, in this last case the authors refer the consideration of a SSQ threshold of 0.17 while using nylon-coated fibers adhered on the surface of the concrete due to the proximity of inaccurate values. 

The low SSQ values are close to the crack location in the segments adhered to the surface of the concrete and within a wider area in the rebar segment. This is explained as segments with large strain gradients increase the noise levels of the DOFS measurements [39].

### 3.2. Overcoming Drop of SSQ

As mentioned before, in order to feasibly analyse the performance of the fiber sensor in the embedded segment FI after the occurrence of cracking it is necessary to extract all the points with an inaccurate SSQ value. Nevertheless, when performing this task and although the manufacturers’ user guide recommends the use of the 0.15 SSQ value as threshold, the authors observed that several isolated peaks still remained in the measured data of segment FI. This is due to the surrounding proximity to the aforementioned values with low SSQ. 

Therefore, it was observed that using a threshold of 0.20 these remaining peaks were optimally eliminated. The authors believe that this threshold is subjected to the specific case of the use of a polyimide-coated fiber in the embedded segment in the concrete.

**Figure 19:**
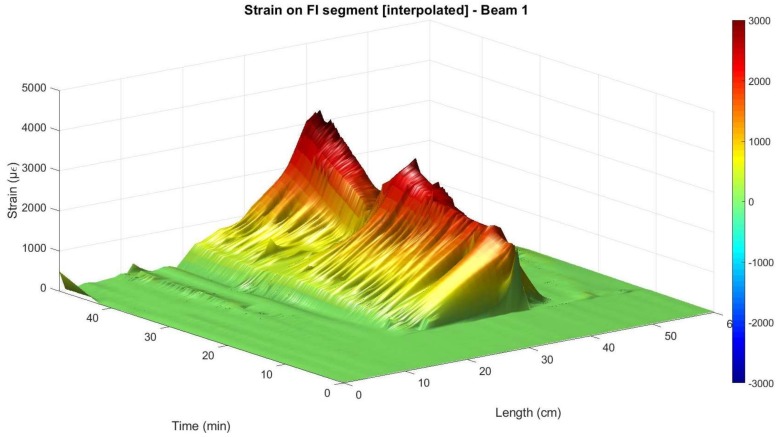
Interpolated measured strain at the embedded segment in Beam 1.

**Figure 20:**
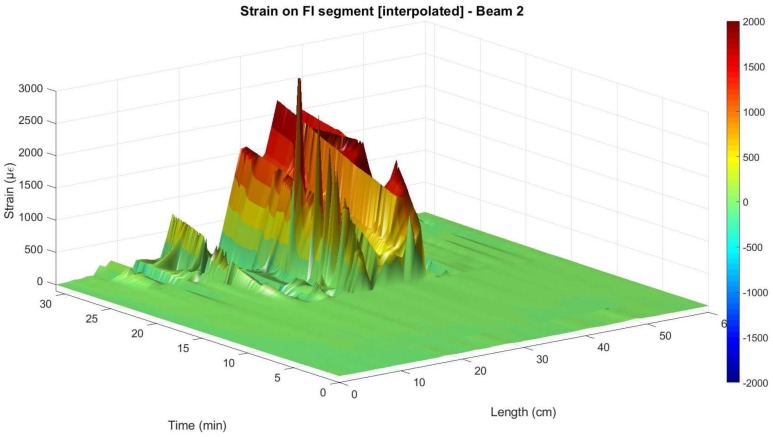
Interpolated measured strain at the embedded segment in Beam 2.

After removing these unreliable data points, it is then possible to interpolate the surface points of the embedded segment in order to better analyse the behavior of the structural element after cracking. The result of the elimination of the values with SSQ lower than 0.2 and posterior interpolation in segment FI is shown in Figure 19 and Figure 20 for Beam 1 and Beam 2, respectively. 

From Figure 20, it is possible to observe that despite the removing of the unreliable values with exactly the same criteria on both beam specimens, after the surface interpolation a reduced number of peaks is still observable for beam 2. This is attributed to the better performance of the cyanoacrylate adhesive as a bonding product when used in the rebar element jointly with a polyimide-coated fiber.

Nevertheless, after this post-processing it is observable how the DOFS data mostly presents strain evolution that is compatible with the applied load. This is especially noticeable in Beam 1 since, as mentioned before, in this specimen the sensor worked properly until a later stage of the loading process. In this specimen and from Figure 19, it is shown how the DOFS interpolated measurements follows the same load cycle of loading, unloading and a new loading until the rupture of the fiber.

**Figure 21:**
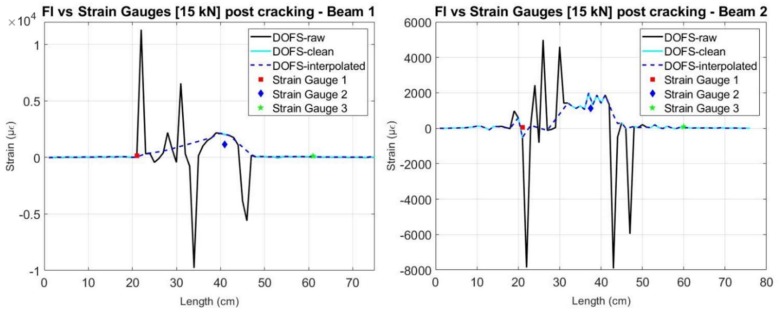
Comparison of the measured values before and after interpolation of the DOFS with the strain gauges for 15 kN—Beam 1 (**left**) and Beam 2 (**right**).

The effect of this post-processing is also represented in Figure 21 for the specific load level of 15 kN for both beams. Here, it is observable how the main inaccurate peaks were removed and the interpolated values got closer to what is measured by the strain gauges and what is expected from a three-point load test in a simply supported element. 

### 3.3. Performance Assessment of the Embedded DOFS

**Figure 22:**
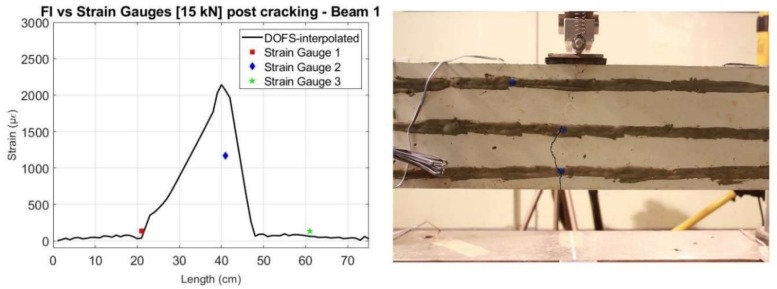
Comparison of interpolated values of DOFS with Strain Gauges for a 15 kN load (**left**) and photo of beam specimen with highlighted crack (**right**)—Beam 1.

**Figure 23:**
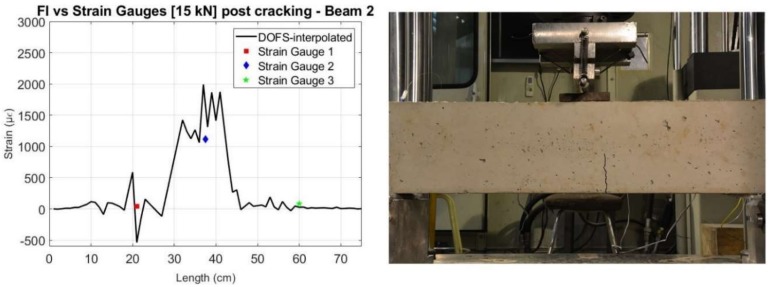
Comparison of interpolated values of DOFS with Strain Gauges for a 15 kN load (**left**) and photo of beam specimen with highlighted crack (**right**)—Beam 2.

When comparing the values after cracking with those measured by the strain gauges, we can see, in general, a good agreement, as shown in Figure 22 and Figure 23 for a load level of 15 kN. Furthermore, it is observable how the crack observed visually directly on the beam specimens for this load level, agrees with the data obtained by the sensors.

**Figure 24:**
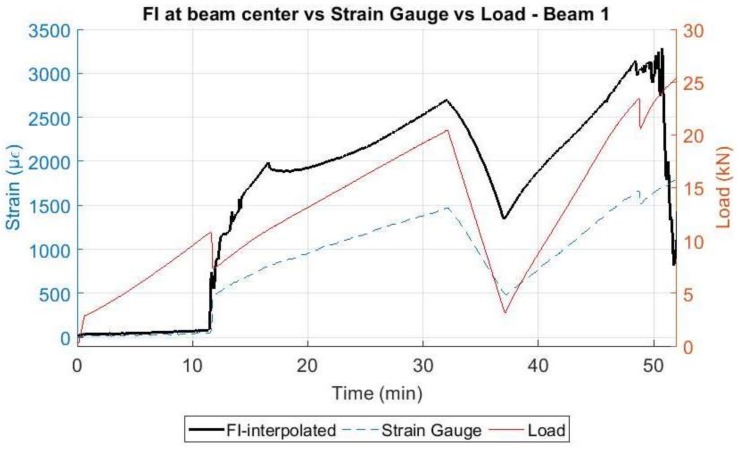
Comparison of DOFS with strain gauge and the applied load—Beam 1.

**Figure 25:**
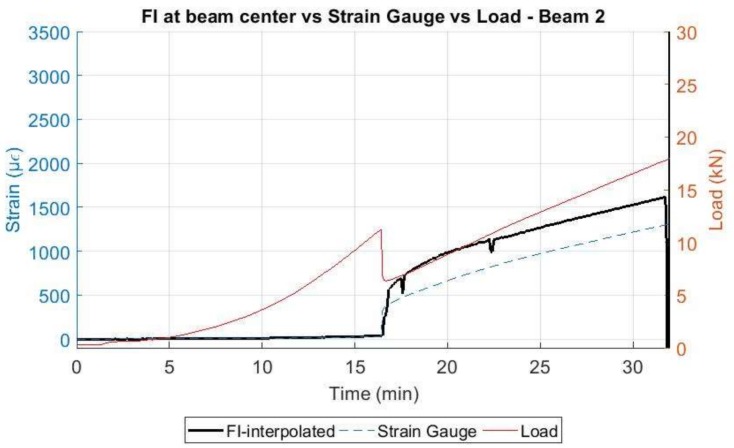
Comparison of DOFS with strain gauge and the applied load—Beam 2.

When comparing the evolution of the strain data obtained by the DOFS at the mid-span point of the rebar and the strain gauge 2 (also located close to the center) with the applied load, Figure 24 and Figure 25 are obtained.

In these figures, it is possible to see how the strain in the rebar increases significantly only when load levels reach the cracking load and how the DOFS measurements follow the same trend as the strain gauge according to the applied load. The difference in the values measured by the DOFS and the strain gauge are explained by two facts: first, the location of the strain gauge does not match exactly the point where the DOFS measurement is represented and, second, as the fiber and the strain gauge are bonded on opposite parts of the rebar, then a local bending effect in the rebar itself may derive on a local compression and local tension to be added to the general tension in the rebar due to the global bending induced by the vertical applied load. It is again observable, how the fiber in beam 1 was able to perform longer until rupturing around minute 51 for a load of 24.3 kN while on beam 2, the fiber ruptured around minute 32 for a load of 17.8 kN.

Despite the challenge presented by the reduction of the SSQ values associated to some points of the DOFS after the occurrence of cracking to levels that implied unreliable measurements, due to the distributed capacities of this sensor technology, it is still possible to gather relevant information corresponding to the cracked behavior of the concrete beams. This is obtained using only one single sensor, where in other scenarios, multiple discrete sensors would be required.

## 4. Conclusions

In this paper a new method of implementation of Rayleigh OFDR-based DOFS in reinforced concrete structures in both uncracked and cracked scenarios is discussed. With the intention of increasing the accuracy of the sensor measurements and to increase its protection to external environmental effects, a thin polyimide-coated distributed sensor was bonded directly to a reinforcing steel bar without any previous mechanization.

This implementation technique was first tested in an isolated S500 steel rebar during a simple tensile load test within the elastic range of this material. Comparing with the results coming from the strain gauges, it was observed how the DOFS provided reliable measurements for the entire length of bonding and duration of the test.

Afterwards, a three-point load test with a loading and unloading sequence after cracking was conducted in two similar reinforced concrete beams, where a 5.2 m DOFS was simultaneously deployed in the rebar and, after the hardening of the concrete, bonded to the outer surface of the beam through different segments intended to measure different structural patterns. Strain gauges were also used for comparison purposes. The only difference between the two beam specimens was the adhesive, cyanoacrylate for beam 1 and a two-component epoxy for beam 2. This adhesive was also used to bond the same DOFS to the concrete surface. The main findings of this study are the following:

1. The optic fiber bonded to the rebar and crossing the cracks delivered good results even in the case of loading and unloading of the specimen, what may create some disturbances in the fiber surrounding the lips of the crack due to the roughness and heterogeneity of the concrete material.
2. The distributed optical fiber sensors were able to detect and locate the formation of cracking at the surface of the concrete and to reflect how while this cracking had already occurred at the concrete surface, this damage was not detected in the rebar element until a later load stage.
3. After a certain load which corresponded to a significant stiffness change in the beam specimens and the detection of damage at the rebar element, some measurements at the location of the damage started to present unreliable data values. This was especially noticeable for the rebar-adhered DOFS segment (FI). This is associated with a decrease of the spectral shift quality (SSQ) values at these point locations.
4. The results show that a minimum spectral shift quality (SSQ) of 0.20 is necessary to get reliable results when embedded polyimide-coated DOFS are used in the reinforcing bars. By now, the only recommendations in this sense were the one by the system acquisition manufacturers’ users guide which refers DOFS data points with SSQ values below 0.15 to be unreliable, and that of Brault et al. [38] which considers SSQ values below 0.17 as unreliable when using nylon-coated fibers bonded to concrete surfaces. In any case, further research is planned on the preventive decrease of the SSQ values by analysing the conditions causing this effect circumstance and the optimal way to avoid them. The proposed threshold of 0.20 for the SSQ value should be also verified in further tests using polyimide-coated DOFS.
5. A method is presented to overcome the low SSQ values with an interpolation post-processing routine that enables a better interpretation of the measurements in later steps of the load test.
6. A better performance was observed when using cyanoacrylate adhesive in the reinforcing steel compared to a two-component epoxy in the case that polyimide-coated DOFS are used. Not only did the embedded cyanoacrylate bonded segment presented smoother data within the uncracked range, but an enhanced performance during a longer time and load level was achieved too.
7. The tests have shown the feasibility of deploying a single polyimide coated Rayleigh OFDR based DOFS, simultaneously to the rebar and external surface of a reinforced concrete beam. This results in a simpler and more economic experimental set-up.

## Figures and Tables

**Table 1:** Mechanical properties of the concrete.

Specimen	$f_{cm}$ (MPa)	$f_{ctm}$(MPa)	*E* (GPa)	$\varepsilon_{fct}$ (µε)
Beam 1	38.41	3.076	28.366	108
Beam 2	44.36	3.523	27.353	129
